# Deep learning-based survival prediction of oral cancer patients

**DOI:** 10.1038/s41598-019-43372-7

**Published:** 2019-05-06

**Authors:** Dong Wook Kim, Sanghoon Lee, Sunmo Kwon, Woong Nam, In-Ho Cha, Hyung Jun Kim

**Affiliations:** 1 Department of Oral & Maxillofacial Surgery, Yonsei University College of Dentistry, 50-1 Yonsei-ro, Seodaemun-gu, Seoul, 03722 Republic of Korea; 2 Oral Cancer Research Institute, Yonsei University College of Dentistry, 50-1 Yonsei-ro, Seodaemun-gu, Seoul, 03722 Republic of Korea; 3 Department of Oral & Maxillofacial Surgery, Dankook University Jukjeon Dental Hospital, 152 Jukjeon-ro, Suji-gu, Yongin-Si, Gyeonggi-do, 16890 Republic of Korea

**Keywords:** Machine learning, Oral cancer, Surgical oncology

## Abstract

The Cox proportional hazards model commonly used to evaluate prognostic variables in survival of cancer patients may be too simplistic to properly predict a cancer patient’s outcome since it assumes that the outcome is a linear combination of covariates. In this retrospective study including 255 patients suitable for analysis who underwent surgical treatment in our department from 2000 to 2017, we applied a deep learning-based survival prediction method in oral squamous cell carcinoma (SCC) patients and validated its performance. Survival prediction using DeepSurv, a deep learning based-survival prediction algorithm, was compared with random survival forest (RSF) and the Cox proportional hazard model (CPH). DeepSurv showed the best performance among the three models, the c-index of the training and testing sets reaching 0.810 and 0.781, respectively, followed by RSF (0.770/0.764), and CPH (0.756/0.694). The performance of DeepSurv steadily improved with added features. Thus, deep learning-based survival prediction may improve prediction accuracy and guide clinicians both in choosing treatment options for better survival and in avoiding unnecessary treatments.

## Introduction

Over 350,000 people worldwide will be diagnosed with oral cancer this year^1^. It will cause over 170,000 deaths, killing roughly one person every 3 minutes^1^. Given its location, its impact on quality of life is quite large and treatment is often challenging. Of those newly diagnosed individuals, only slightly more than half will survive after 5 years. This number has not significantly improved in past few decades, despite advances in diagnostic techniques and state-of-the-art treatment modalities^2^.

Treatment of oral cancer depends on the staging system, and inaccurate staging system may lead to insufficient or unnecessary treatment. While various prognostic markers and therapeutic targets have been proposed in recent decades, they are not reflected in the current staging system^3–7^. This may partly account for the unchanged overall prognosis of oral cancer in the recent decades^8^.

The log-rank test and Cox proportional hazard (CPH) model are the most frequently used methods for survival analyses of cancer patients. The CPH model is used to identify the prognostic factors that significantly affect the survival of cancer patients. However, as it assumes that the outcome is a linear combination of covariates, it may be too simplistic to properly predict cancer patient outcomes, which seem complex and involve interactions between variables. The hazard function at time *t* for subject *i* with covariates *x* can be expressed as shown in (1). Moreover, this model does not provide a decision rule to be used in clinical practice.

1 $$\mathrm{ln}\,\frac{{h}_{i}(t)}{{h}_{0}(t)}={\beta }_{1}{x}_{i1}+{\beta }_{2}{x}_{i2}+{\beta }_{3}x{i}_{3}+\cdots +{\beta }_{k}{x}_{ik}$$

There have thus been attempts to accurately predict cancer patients’ survival, including in the field of oral cancer. One approach is the nomogram. In a study based on 96 patients, Kim *et al*. constructed a nomogram for predicting the survival of oral SCC patients using clinical variables and molecular markers IMP3 and p53^8,9^.

Machine learning, a branch of artificial intelligence which enables detection of relationships from complex datasets, has recently been employed for this purpose. Previous studies applying machine learning to oral cancer have reported good results. Shams *et al*. used machine learning with gene expression profiling to predict the possibility of oral cancer development in terms of the malignant transformation of oral premalignant lesions^10^. The study was conducted on 86 patients, 51 of whom developed oral cancer and 31 remained cancer free. Deep learning along with support vector machine and other methods were compared. Highest accuracy was achieved when deep learning was applied with the Fisher discriminant analysis, achieving 96.5%, 98.1%, and 94.2% for accuracy, sensitivity, and specificity. This can be considered an improvement compared to previous results using traditional statistical methods, which showed a misclassification rate of 16%, with 91% sensitivity and 76% specificity^11^.

Kann *et al*. utilized deep learning to predict ENE before surgery using 270 head and neck cancer patients’ CT data^12^. Among them, oral SCC patients accounted for 106. ENE, also known as extracapsular extension or extracapsular spread, is known to be associated with higher rates of recurrence and poorer survival. Currently, ENE can only be diagnosed from postoperative pathology. While previous studies have reported an area under a receiver operating characteristic curve (AUC) ranging from 0.65–0.694, Kann *et al*. reported an AUC of 0.91, thus showing the potential of the deep learning model for use as a clinical decision-making tool to help guide head and neck cancer patient management.

However, when it comes to the prediction of cancer survival, accuracy and AUCs cannot sufficiently characterize the outcome. Cancer survival cannot be described only with binary data (survival and death) but should incorporate ‘time to event’ as well. Chang *et al*. reported 93.8% accuracy with AUC of 0.90 in predicting oral cancer patients’ prognosis^13^. The study was based on 31 oral cancer patients’ clinicopathologic status and genomic markers. Due to the small sample size, the result was based on 5-fold cross-validation. Accuracy and AUC were measured in terms of disease status at a particular time point, and the time to event element was not taken into account. Though its results were not based on survival analysis, it is representative of early reports showing the favorable performance of machine learning with a relatively small dataset.

Tseng *et al*. conducted a study applying machine learning to oral cancer prognosis prediction of clinicopathologic features of 674 patients^14^. Implemented methods were decision tree and artificial neural network, which now constitute primitive forms of random forest and deep learning. The study did not consider the time to event nature and only estimated disease status at the 5^th^ year. Reported accuracy of training set and testing set was 98.4% and 93.9%. Though it was not a survival analysis considering the time element, it was based on the largest oral cancer patient dataset to date, and is a notable early attempt to apply machine learning to oral cancer survival prediction.

For binary data, such as presence or absence of a disease, area under the receiver operating curve (AUC) can be used to estimate the performance of a model. However, in addition to binary disease status, cancer survival analysis must take into account time to event. Harrell’s c-index is known to be the most accurate and suitable method for estimating prediction error^15^. The c-index is used most commonly as a metric for survival prediction and reflects a measure of how well a model predicts the ordering of patients’ death times. A c = 0.5 is the average of a random model, and c = 1 refers to a perfect match of death time ranking^15,16^.

To our knowledge, this study in oral cancer survival implementing a recently-developed machine learning technique utilizes the largest dataset of its kind while taking time to event into account. We implemented random survival forests and deep learning to predict the survival of oral squamous cell carcinoma (SCC) patients, who comprise 90% of oral cancer patients^2,16,17^. Deep learning based-survival model, random survival forest (RSF), and CPH model were built and their performance compared with one another using Harrell’s c-index.

## Results

### Clinical characteristics

**Figure 1:**
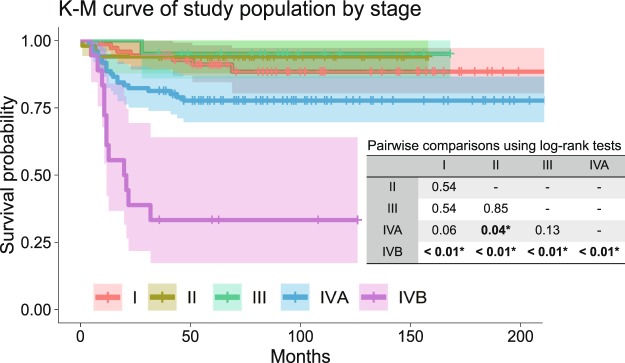
Kaplan-Meier curve by stage of overall dataset, with pairwise comparisons using log-rank test. Statistically significant survival differences were only noted between stages IVB & others, and between IVA & II.

Of the 255 patients’ records suitable for analysis, 141 patients were in stages I, II, and III, and 114 patients were in stage IV, according to the American Joint Committee on Cancer (AJCC) 8^th^ cancer staging manual. Among them, 65 patients had loco-regional recurrence and 44 patients died due to cancer-related causes. The mean follow-up period was 80.5 months. The clinical characteristics of the dataset is shown in Table 1, and Kaplan-Meier survival curve by TNM stage is shown in Fig. 1. There were no statistically significant survival differences among stages I, II and III. Statistically significant survival differences were only noted between stages IVB & others, and between IVA & II (Fig. 1).

**Table 1:** Clinical characteristics of the overall dataset.

Total number of patients		n = 255
Sex (%)	Female	86 (33.7)
	Male	169 (66.3)
Age (mean (SD))		57.6 (12.13)
Site (%)	Tongue	73 (28.6)
	Mandibular gingiva	62 (24.3)
	Maxillary gingiva	40 (15.7)
	Buccal cheek	36 (14.1)
	Retromolar trigone	25 (9.8)
	Floor of mouth	18 (7.1)
	Lip	1 (0.4)
Histologic grade (%)	Well differentiated	72 (28.2)
	Moderately differentiated	144 (56.5)
	Poorly differentiated	39 (15.3)
TNM Stage^†^ (%)	I	71 (27.8)
	II	50 (19.6)
	III	20 (7.8)
	IVA	96 (37.6)
	IVB	18 (7.1)
T stage^†^ (%)	T1	79 (31.0)
	T2	65 (25.5)
	T3	22 (8.6)
	T4a	86 (33.7)
	T4b	3 (1.2)
N stage^†^ (%)	N0	105 (41.2)
	N1	16 (6.3)
	N2a	12 (4.7)
	N2b	23 (9.0)
	N2c	17 (6.7)
	N3b	82 (32.2)
	Nx^‡^	79 (31.0)
Bone marrow invasion (%)		58 (22.7)
Perineural invasion (%)		15 (5.9)
Lymphovascular permeation (%)		17 (6.7)
Presence of tumor at resection margin (%)		36 (14.1)
Extranodal extension (%)		29 (11.4)
Postoperative radiation therapy (%)		86 (33.7)
Postoperative CCRT^§^ (%)		17 (6.7)
Overall recurrence (%)		65 (25.5)
Cancer-related death (%)		44 (17.3)
Follow-up months (mean (SD))		80.5 (52.02)

^†^According to AJCC 8th classification.

^§^Concurrent chemoradiotherapy.

**Figure 2:**
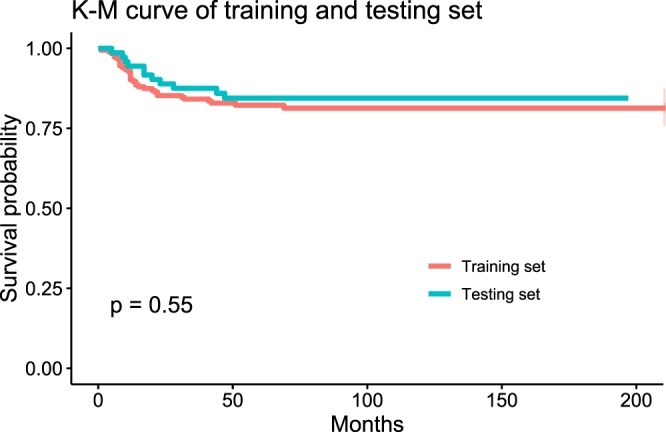
Kaplan-Meier curve of training and testing sets. There was no statistically significant difference between the survival of training and testing sets in log-rank test (p = 0.55).

The entire dataset was split into two mutually exclusive datasets, 70% into the training set and 30% into the testing set. The training set was utilized to generate the prediction model and the remaining 30% was employed to estimate the model’s accuracy. There were no statistically significant differences in the features between the two sets (Table2). Difference in survival outcome was absent between the two sets as well (Fig. 2).

**Table 2:** Characteristics of training and testing sets.

		Training set	Testing set	p-value
		(n = 183)	(n = 72)	
Sex	Female	63 (34.4%)	23 (31.9%)	0.818
	Male	120 (65.6%)	49 (68.1%)	
Age (mean(SD))		57.3 (12.6)	58.3 (11.0)	0.830
Site	Tongue	53 (29.0%)	20 (27.8%)	0.407
	Mandibular gingiva	50 (27.3%)	12 (16.7%)	
	Maxillary gingiva	28 (15.3%)	12 (16.7%)	
	Buccal cheek	24 (13.1%)	12 (16.7%)	
	Retromolar trigone	17 (9.3%)	8 (11.1%)	
	Floor of mouth	10 (5.5%)	8 (11.1%)	
	Lip	1 (0.5%)	0 (0.0%)	
Histologic grade	Well-differentiated	59 (32.2%)	13 (18.1%)	0.476
	Moderately-differentiated	92 (50.3%)	52 (72.2%)	
	Poorly-differentiated	32 (17.5%)	7 (9.7%)	
TNM Stage	I	50 (27.3%)	21 (29.2%)	0.995
	II	37 (20.2%)	13 (18.1%)	
	III	14 (7.7%)	6 (8.3%)	
	IVA	70 (38.3%)	26 (36.1%)	
	IVB	12 (6.6%)	6 (8.3%)	
T stage	T1	57 (31.1%)	22 (30.6%)	0.912
	T2	47 (25.7%)	18 (25.0%)	
	T3	13 (7.1%)	9 (12.5%)	
	T4a	64 (35.0%)	22 (30.6%)	
	T4b	2 (1.1%)	1 (1.4%)	
N stage	Nx	72 (39.3%)	33 (45.8%)	0.422
	N0	13 (7.1%)	3 (4.2%)	
	N1	8 (4.4%)	4 (5.6%)	
	N2a	17 (9.3%)	6 (8.3%)	
	N2b	11 (6.0%)	6 (8.3%)	
	N3b	62 (33.9%)	20 (27.8%)	
Bone marrow invasion	Absence	142 (77.6%)	55 (76.4%)	0.967
	Presence	41 (22.4%)	17 (23.6%)	
Perineural invasion	Absence	175 (95.6%)	65 (90.3%)	0.137
	Presence	8 (4.4%)	7 (9.7%)	
Lymphovascular permeation	Absence	174 (95.1%)	64 (88.9%)	0.094
	Presence	9 (4.9%)	8 (11.1%)	
Resection margin	Free from tumor	158 (86.3%)	61 (84.7%)	0.893
	Presence of tumor	25 (13.7%)	11 (15.3%)	
Extranodal extension	Absence	164 (89.6%)	62 (86.1%)	0.565
	Presence	19 (10.4%)	10 (13.9%)	
Postoperative RT	No	122 (66.7%)	47 (65.3%)	0.949
	Yes	61 (33.3%)	25 (34.7%)	
Postoperative CCRT	No	169 (92.3)	69 (95.8)	0.411
	Yes	14 (7.7)	3 (4.2)	
Overall Recurrence^†^	No	136 (74.3)	54 (75.0)	1.000
	Yes	47 (25.7)	18 (25.0)	
Cancer related death	No	150 (82.0)	61 (84.7)	0.734
	Yes	33 (18.0)	11 (15.3)	
Follow-up months (mean (sd))		79.7 (53.7)	82.3 (47.9)	0.448

The entire dataset was split into two groups, 70% for training and 30% for testing. There were no statistically significant differences among the features of the two groups. Mann-Whitney U test for continuous variables; Chi square test, Fisher’s exact test, and Cochran-Armitage Trend test for categorical variables.

RT = Radiation therapy, CCRT = Concurrent chemoradiotherapy.

^†^Includes local recurrences and regional recurrences.

### Comparing the performance of deep-learning based survival prediction with random survival forest (RSF) and Cox proportional hazard (CPH) model

**Figure 3:**
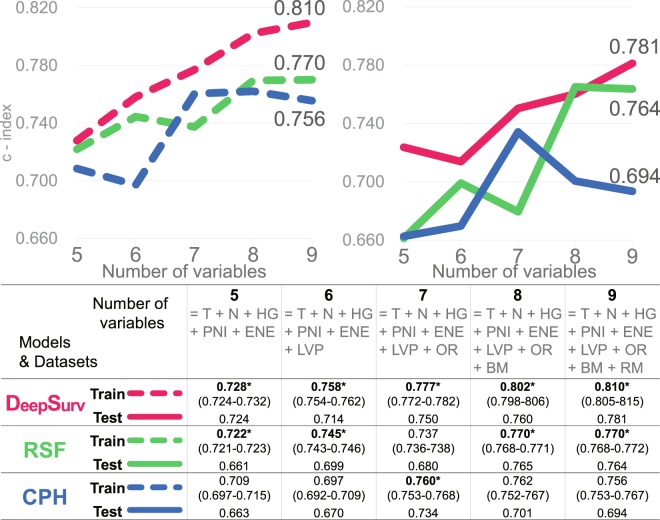
Performance of DeepSurv, RSF, and CPH model in terms of c-index (95% confidence interval). DeepSurv performed best among the three models, showing a relatively more upward trend. Points 6, 8, and 9 indicate where statistically insignificant factors (LNP, BM and RM) were added. (T = T stage, N = N stage, HG = Histologic grade, PNI = Perineural invasion, ENE = Extranodal extension, LVP = Lymphovascular permeation, OR = Overall recurrence, BM = Bone marrow invasion, RM = Presence of tumor at resection margin, RSF = Random survival forest, CPH = Cox proportional hazard model, OOB = Out-of-bag).

Survival models based on DeepSurv^16^ (a deep learning-based model), Random survival forest (RSF)^17^, and a model based on CPH regression were built with the training set. The performance of these three models were compared by calculating Harrell’s c-index, which measures the concordance between predicted risks and actual survival, applied to both the training and testing set^15^ (Fig. 3). DeepSurv performed best among the three models, the c-index on training and testing sets reaching 0.810 and 0.781, followed by RSF (0.770/0.764), and the CPH model (0.756/0.694). The results from different random splits of the dataset yielded consistent results (Supplementary Figs S1–S4).

**Figure 4:**
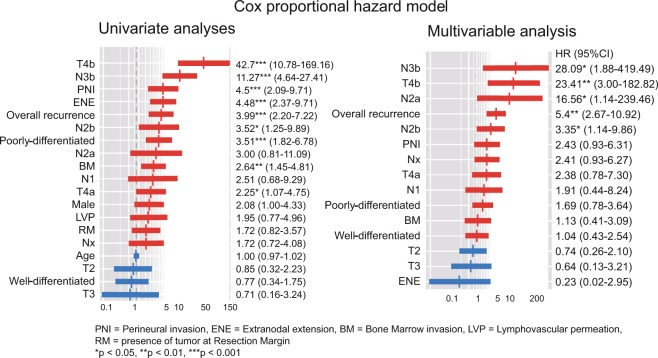
Univariate & Multivariable CPH analyses. Variables are sorted in descending order of hazard ratio. Advanced T stage, N stage, perineural invasion (PNI), extranodal extension (ENE), overall recurrence, poorly differentiated histologic grade (HG), and bone marrow invasion (BM) were significant features in univariate analyses and were taken into multivariable analysis. Advanced T, N stage and overall recurrence remained statistically significant in multivariable analysis.

Starting with five, the features were incrementally added up to nine. The first five features were statistically significant variables in univariate CPH regression (Fig. 4). Statistically insignificant and significant features were subsequently added. As the features were added one after another, the c-index of DeepSurv showed a relatively steadier upward trend, while RSF and CPH models showed decreases at the points where statistically insignificant features (LVP, BM, and RM) were added (Fig. 3). Though these variables turned out to be statistically insignificant in CPH analysis, they are still considered important in decision making and prognosis in a clinical setting^18,19^.

### Cox proportional hazard (CPH) model

The CPH model was built with the training set. Statistically significant variables in univariate analyses were taken into multivariable analysis. While advanced T stage, N stage, perineural invasion (PNI), extranodal extension (ENE), overall recurrence, poorly differentiated histologic grade (HG), and bone marrow invasion (BM) significantly affected oral SCC patients’ survival in univariate analyses, only the advanced T, N stage and overall recurrence remained statistically significant in multivariable analysis (Fig. 4).

Prediction accuracy was measured by means of c-index on the training and testing sets. As the number of features used to build the model increased from 5 to 9, the c-index did not steadily increase although there were decreases at certain points at which statistically insignificant features were added (Fig. 3). The c-index eventually reached 0.756 and 0.694 for the training and testing sets, which was the lowest among the models (Fig. 3).

### Random survival forest (RSF)

**Figure 5:**
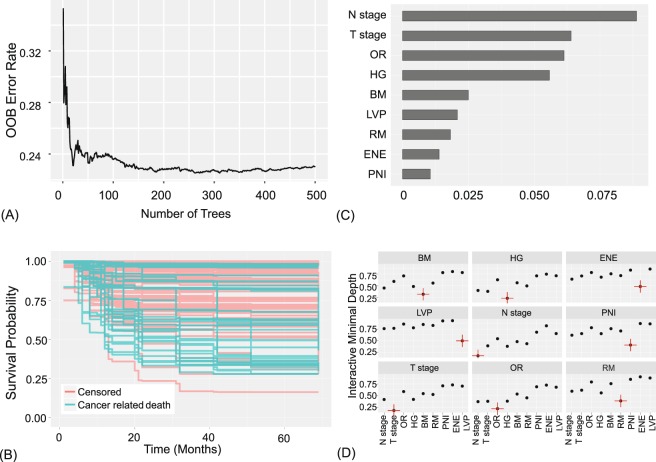
Random survival forest model. 9 features were used to construct the model: T stage, N stage, histologic grade (HG), perineural invasion (PNI), extranodal extension (ENE), lymphovascular permeation (LVP), overall recurrence (OR), bone marrow invasion (BM), presence of tumor at resection margin (RM). (**A**) OOB error rates. (**B**) Estimated survival of testing set. (**C**) Variable importance plot. Higher VIMP indicates the variable contributes more to predictive accuracy. (**D**) Variable interaction plot. Lower values indicate higher interactivity, with the target variable marked in red. T stage and N stage show relatively higher interactions with other variables.

Prediction error is calculated using OOB data (training set), and also using the testing set (Fig. 5A,B). The variable importance (VIMP), shown in Fig. 5C, was obtained by measuring the decrease in prediction accuracy when randomizing a particular variable^17,20^. Higher VIMP indicates the variable contributes more to predictive accuracy^21^. Note that the three highest ranking variables by VIMP match those selected by the multivariable CPH in Fig. 4.

Interactions between variables are measured and displayed in Fig. 5D and Supplementary Table S5 in pairwise manner^15^. It can be said that there are interactions between the two variables if a split on one variable in a tree makes a split on another variable more or less possible^22,23^. An interaction is measured based on minimal depth, defined as the distance from the root node to the node where a variable first splits^22^. T and N stages showed lowest minimal depth and are expected to be associated with other variables.

As the number of variables used to build the model increased from 5 to 9, there were increases and decreases in the c-index, eventually reaching 0.770 and 0.764 for the training set and testing set, respectively (Fig. 3).

### DeepSurv

**Figure 6:**
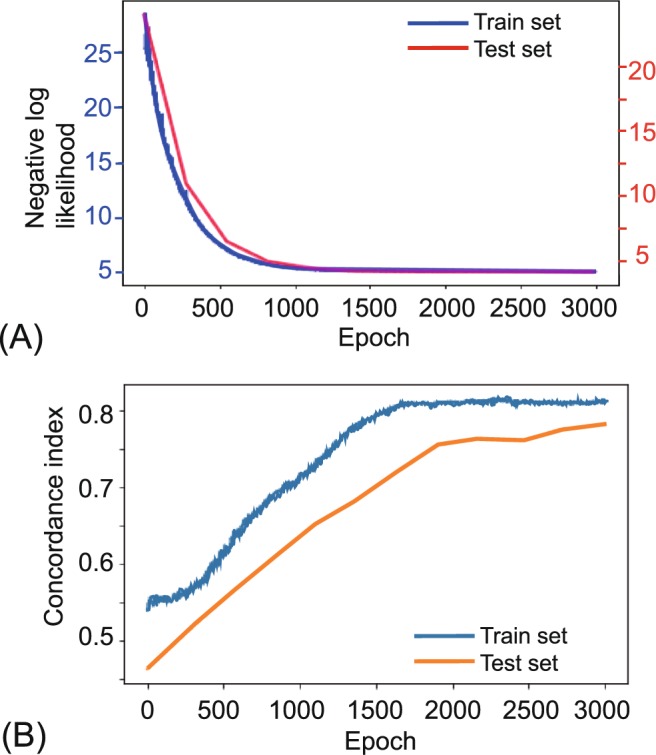
Training and testing history of DeepSurv. The above figure shows the results with 9 features: T stage, N stage, HG, PNI, ENE, LVP, OR, BM, and RM. (**A**) A plot of loss on training and testing sets. The error over each iteration gradually decreased in both the training and testing sets. (**B**) A plot of accuracy. The accuracy in terms of c-index is plotted over each epoch. It does not seem overfitted or underfitted.

The learning process of DeepSurv, a deep learning-based survival prediction is visualized in Fig. 6, showing good fit. The c-index increased more steadily than did CPH and RSF as the number of features to build the model increased (Fig. 3). The c-index of DeepSurv eventually reached 0.810 and 0.781 for the training set and testing set, the highest among the models (Fig. 3).

## Discussion

Traditional hazards-based models such as CPH are not designed to predict an outcome, but to infer variables’ impact on a survival curve. Thus if one wants to predict something like “days till occurrence”, CPH may not be advisable and one should consider a method such as machine learning. While traditional statistics are about explanation, machine learning is about predictions. Traditional statistics may provide good reasons to enroll a patient into a new clinical trial. Machine learning may predict what type of treatment or clinical trial will be most beneficial for a patient by considering a vast amount of information including disease status and genetic profiles. When modeling nonlinear gene interactions, we cannot assume the data satisfies the linear proportional hazards condition, and the CPH model cannot be applied for such purpose. In oral SCC, even the clinical parameters are interrelated. T staging itself includes bone marrow invasion, and N staging considers the presence of extranodal extension according to the AJCC 8th cancer staging manual. Since N stage has a high correlation with ENE, the effect of ENE on survival will split between the two variables and hence get diluted. This effect, known as multicollinearity, becomes problematic when we try to incorporate novel prognostic factors, such as a certain mutation or molecular marker in combination with clinico-pathologic status for predictions. Novel prognostic factors should be independent from pre-existing features to achieve optimized results, or should be powerful enough to be used alone in traditional statistics.

However, certain machine learning algorithms are impervious to problems of this nature. Strong collinearity between variables doesn’t impair the predictive accuracy. Algorithms that internally perform any form of feature selection and are good with high dimensional data are robust against multicollinearity^24–26^. Basically, the fact that we don’t check for multicollinearity in machine learning techniques isn’t a consequence of the algorithm, but rather of the goal. RSF has shown its ability to outperform classic CPH regressions^17,22,23,27,28^. Previous studies applying neural networks failed to demonstrate improvements beyond the classic linear CPH model until Katzman *et al*. recently showed deep neural networks outperforming standard survival analysis^16,29,30^. One of the advantages of a deep learning-based neural network is that it discerns relationships without prior feature selection^16^.

A previous study using nomogram to predict the survival of oral SCC patients using clinical variables and molecular markers of 96 patients yielded a c-index of 0.697, a result comparable to the CPH result in this study; RSF and the deep learning based-model yielded further increases.

The advantage of c-index as a measure of survival performance is that it does not depend on a single fixed time for evaluation. The c-index also specifically accounts for censoring. Furthermore, if c-index is only measured with the training set, overfitting, whereby a model corresponds too exactly to a training set and therefore fails to fit the testing data, cannot be excluded. The c-index in this study was measured on two mutually exclusive datasets, training and testing, and no overfitting was observed. Random forest is known not to overfit^20^. The deep learning-based model in this study showed neither overfit nor underfit, but rather appropriate fit (Fig. 6). We also tried this on different splits of the dataset into training and testing set. The results from each splits were similar to above showing higher performance and improvement with added features in DeepSurv and RSF, compared to CPH.

This study demonstrates that deep learning-based survival predictions show higher performance with oral SCC patient data compared to the classic statistical method. This can benefit patients by stratifying risks and guiding treatment options to save more lives, as well as by avoiding ineffective/unnecessary treatments. Patients will soon benefit from these new techniques, though only if the clinicians learn and apply them. Though this study built the models and validated their performance with mutually exclusive training and testing datasets, we concede that these results are based on a single institution. A larger dataset from multiple centers may improve on these results and further establish the validity of deep learning-based survival prediction in oral cancer patients.

## Methods

### Study participants (acquisition of data)

Medical records of patients who had undergone surgical treatment of oral SCC in our department from January 2000 to November 2018 were retrospectively reviewed. Data of 444 patients were obtained at this step. Patients with metastatic disease, secondary primary cancer, perioperative mortality, a history of previous radiotherapy or/and chemotherapy, or a history of previous head and neck cancer were excluded. Patients with a follow-up period shorter than 36 months were also excluded. After excluding 189 for insufficient data or meeting the exclusion criteria, a total of 255 patients’ records were suitable for analysis. The study was approved by the Ethics Review Board of Yonsei University Dental Hospital Institutional Review Board (IRB No. 2-2018-0035). Written or verbal informed consent was not obtained from any participants because the IRB waived the need for individual informed consent, as this study had a non-interventional retrospective design and all data were analyzed anonymously. The dataset analyzed during the current study are not publicly available but are available from the corresponding author on reasonable request.

### Statistical analysis

The statistical analysis was performed using the R programming language (R Core Team, Vienna, Austria, 2018). Baseline differences between the training set and testing set were assessed using the Mann-Whitney U test for continuous variables, Chi square test, Fisher’s exact test, and Cochran-Armitage Trend test for categorical variables. Survival curves were plotted using the Kaplan-Meier method and compared using log-rank test. To estimate the prognostic effect of the features, univariate and multiple CPH regression analysis were done as well. The moonBook package was used to visualize CPH regression analyses. *p* < 0.05 was considered significant.

### Modelling process

Prior to constructing machine learning models, the data set was split into two mutually exclusive sets. 70% of the overall dataset was assigned as the training set, which was utilized to generate the prediction model. The remaining 30% of the data was designated as the testing set, for use in estimating the model’s accuracy. Harrell’s c-index was used to compare the performance of the proposed methods^15^.

### Random survival forest (RSF)

While statistical methods such as classification and regression trees may be intuitive for clinicians, they suffer from high variance and poor performance^27,31^. These are addressed by random forest, which builds hundreds of trees and outputs the results by voting^20^. RSF reduces variance and bias by using all variables collected and by automatically assessing nonlinear effects and complex interactions^17^. This approach is fully non-parametric, including the effects of the treatments and predictor variables, whereas traditional methods such as CPH utilize a linear combination of attributes^17,22,27^. Random survival forest models were trained using the RandomForestSRC R package.

### Deep learning-based survival analysis

DeepSurv by Katzman *et al*. was implemented as an open-source Python module (https://github.com/jaredleekatzman/DeepSurv)^16^. DeepSurv is a multi-layer feed forward network, of which the output is a negative log partial likelihood, parameterized by the weights of the network. It is implemented in Theano with the Python package Lasagne. It also includes hyper-parameter optimization search. The source code is available at the above URL.

## Supplementary information

Supplementary information
