# Supplementary material for: Deep learning-based survival prediction of oral cancer patients
Source: Sci Rep. 2019 May 6;9:6994. doi: 10.1038/s41598-019-43372-7 (PMC6502856; doi:10.1038/s41598-019-43372-7)
Supplement: Supplementary file 1 — Supplementary information [file 41598_2019_43372_MOESM1_ESM.docx]

**Supplementary Information**

**Title:** Deep learning-based survival prediction of oral cancer patients.

**Authors:** Dong Wook Kim1, Sanghoon Lee1, Sunmo Kwon1, Woong Nam1, 2, In-Ho Cha1, 2, Hyung Jun Kim1, 2*

**Affiliations:**

1 Department of Oral & Maxillofacial Surgery, Yonsei University College of Dentistry, 50-1 Yonsei-ro, Seodaemun-gu, Seoul, 03722, Republic of Korea

2 Oral Cancer Research Institute, Yonsei University College of Dentistry, 50-1 Yonsei-ro, Seodaemun-gu, Seoul, 03722, Republic of Korea

Supplementary Figure **S1. Average of c-index among additional splits.** The variance among different splits S2-S4 implies the prediction error, which indicates ‘what performance one may get from a different dataset,’ in addition to the variance of model performance itself. The variance of performance is shown in Figure 3 as 95% confidence interval, which is obtained from the bootstraps of the training set.


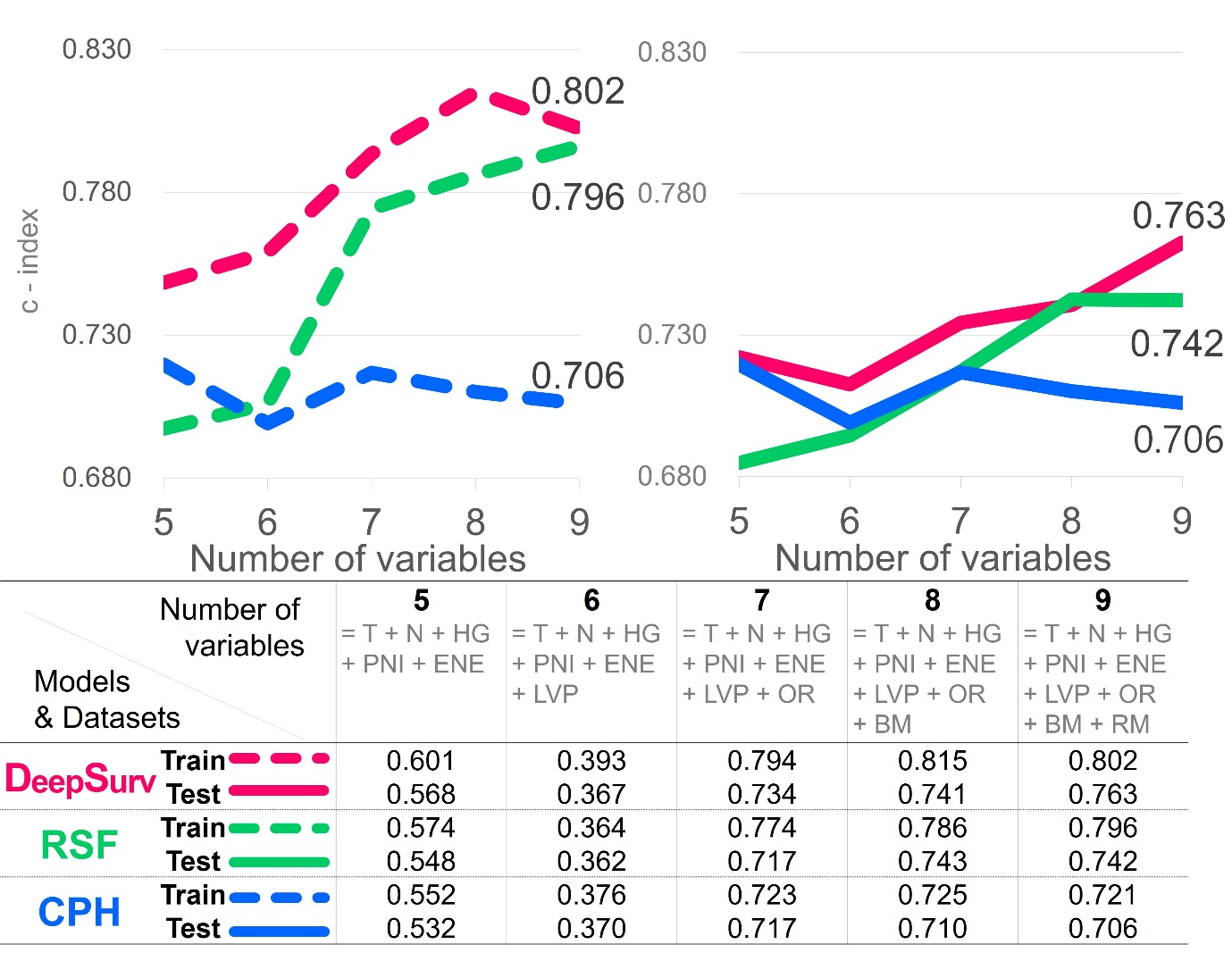


Supplementary Figure **S2. C-index of additional split #1**

**
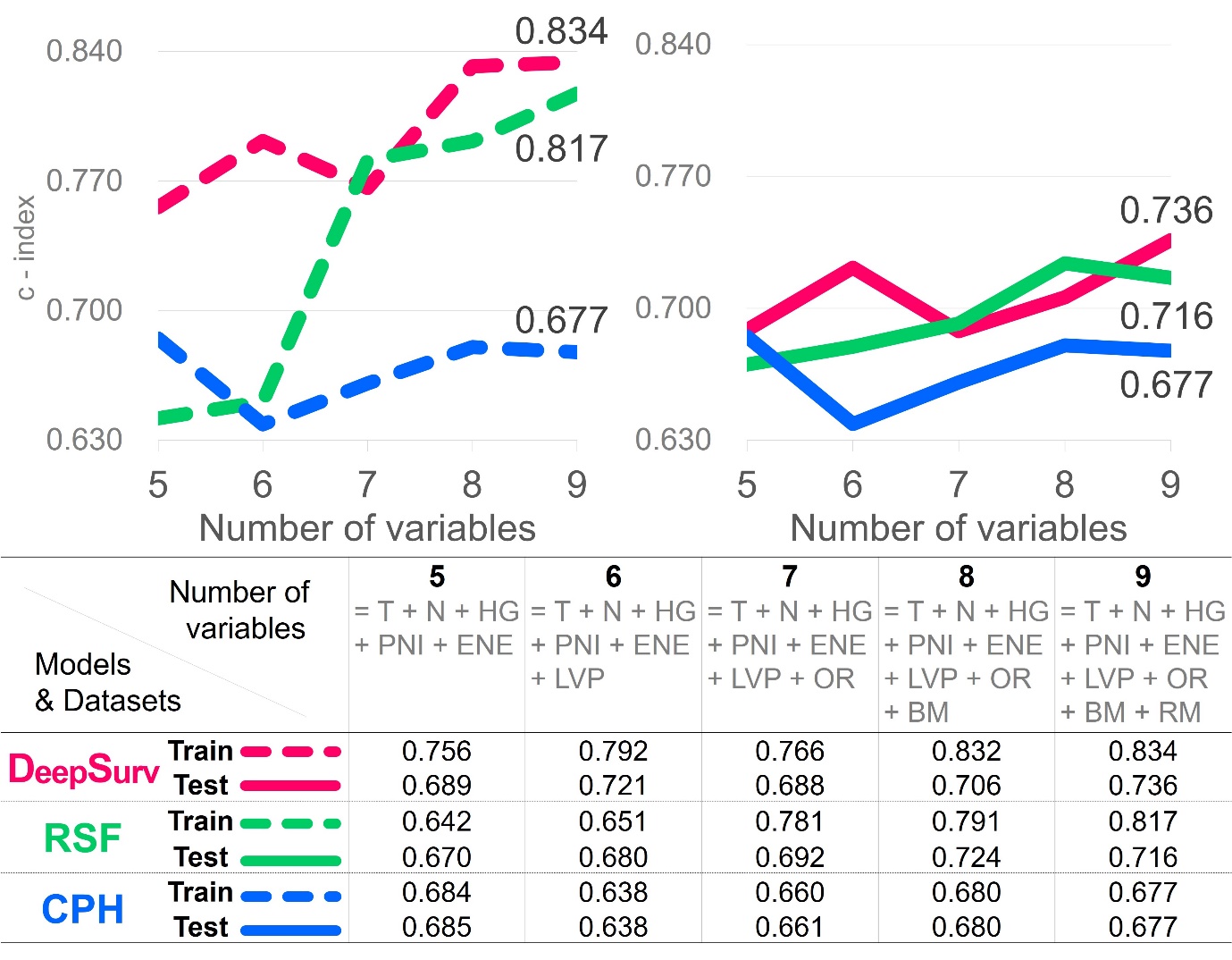
**

Supplementary Figure **S3. C-index of additional split #2**

**
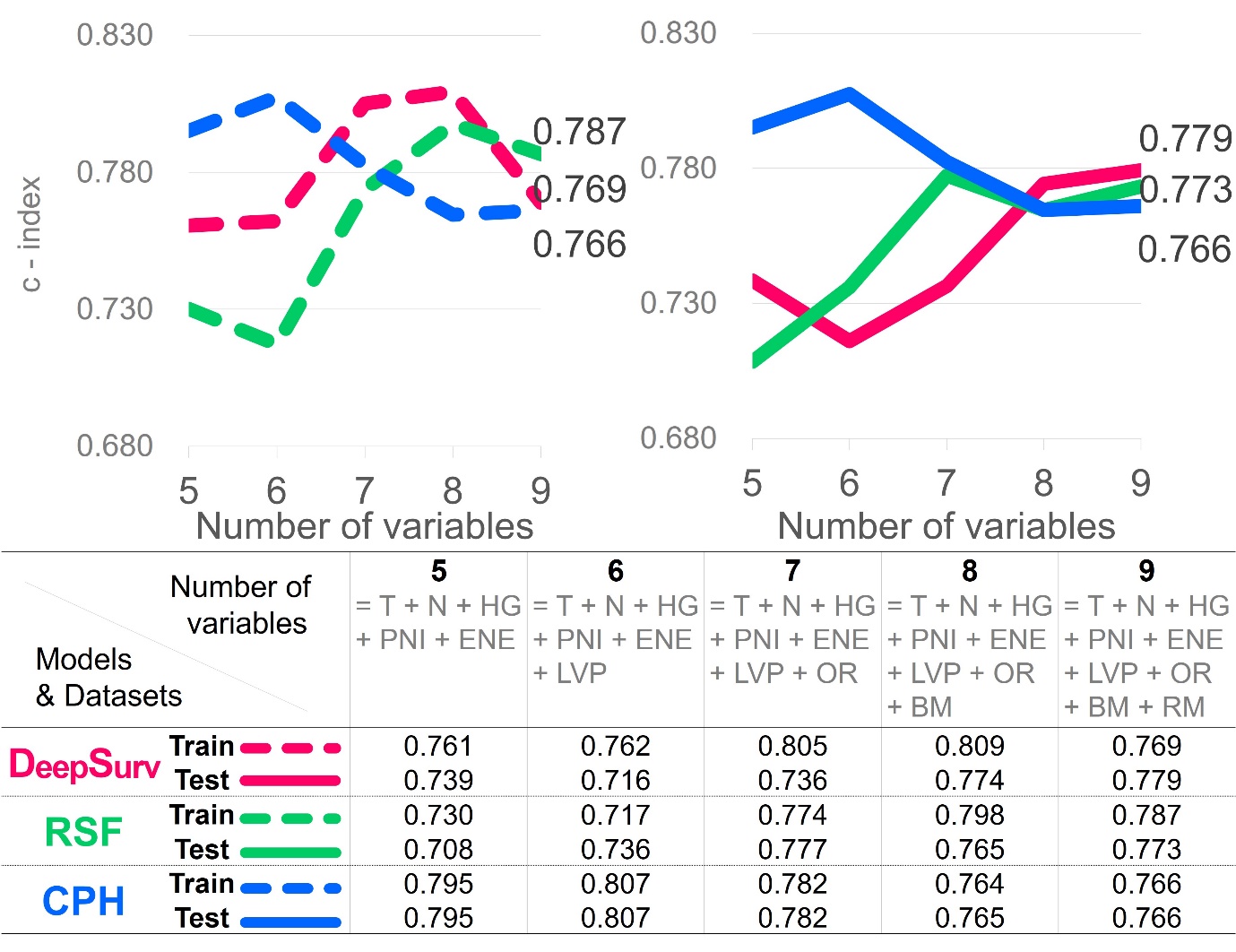
**

Supplementary Figure **S4. C-index of additional split #3**


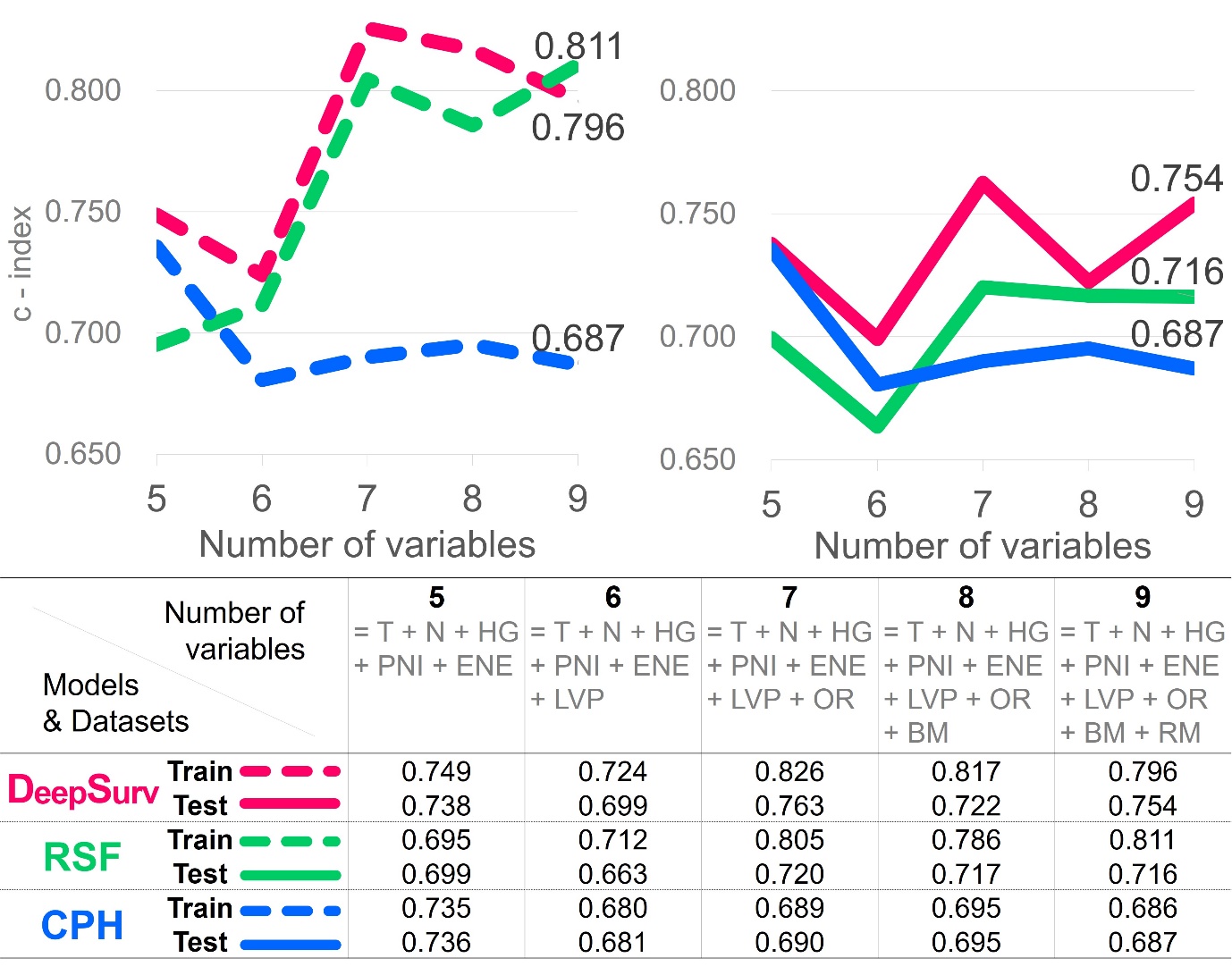


Supplementary Table **S5**. Pairwise minimal depth interactions among variables in random survival forest.

|  | **N stage** | **T stage** | **OR** | **HG** | **BM** | **RM** | **PNI** | **ENE** | **LVP** |
| --- | --- | --- | --- | --- | --- | --- | --- | --- | --- |
| **N stage** | 0.176 | 0.389 | 0.542 | 0.379 | 0.480 | 0.435 | 0.682 | 0.820 | 0.653 |
| **T stage** | 0.418 | 0.183 | 0.588 | 0.420 | 0.544 | 0.526 | 0.711 | 0.731 | 0.705 |
| **OR** | 0.372 | 0.379 | 0.221 | 0.382 | 0.535 | 0.453 | 0.691 | 0.727 | 0.682 |
| **HG** | 0.398 | 0.378 | 0.627 | 0.230 | 0.563 | 0.495 | 0.711 | 0.748 | 0.713 |
| **BM** | 0.450 | 0.592 | 0.710 | 0.483 | 0.317 | 0.556 | 0.781 | 0.803 | 0.782 |
| **RM** | 0.596 | 0.617 | 0.794 | 0.559 | 0.758 | 0.388 | 0.851 | 0.904 | 0.879 |
| **PNI** | 0.617 | 0.652 | 0.778 | 0.651 | 0.758 | 0.709 | 0.407 | 0.868 | 0.861 |
| **ENE** | 0.641 | 0.709 | 0.783 | 0.683 | 0.755 | 0.716 | 0.834 | 0.483 | 0.857 |
| **LVP** | 0.759 | 0.766 | 0.857 | 0.776 | 0.848 | 0.827 | 0.925 | 0.930 | 0.497 |
| OR = Overall recurrence, HG = Histologic grade, PNI = Perineural invasion, ENE = Extranodal extension , LVP = Lymphovascular permeation, OR = Overall recurrence, BM = Bone marrow invasion, RM = Resection margin | | | | | | | | | |
